# Epoxy/BaTiO_3_ Nanocomposites: Tunable Electrical Conductivity and Engineering-Applicable Insulation, Thermal, and Mechanical Properties

**DOI:** 10.3390/ma19101975

**Published:** 2026-05-11

**Authors:** Huize Cui, Han Wang, Wenwen Gu, Chumeng Luo, Yan Zhang, Chuang Zhang, Shengtao Li

**Affiliations:** 1 China Electric Power Research Institute, Beijing 100192, China; jameschz@126.com (H.C.);; 2 School of Electrical Engineering, Xi’an Jiaotong University, Xi’an 710049, China; handsomewh@stu.xjtu.edu.cn; 3 State Grid Jilin Electric Power Co., Ltd., Electric Power Research Institute, Changchun 130021, China

**Keywords:** epoxy composites, tunable electrical conductivity, encapsulation, IGBT, electric field distribution

## Abstract

Epoxy/BaTiO_3_ nanocomposites with varying filler contents of BaTiO_3_ were prepared and characterized for flexible DC insulation applications such as IGBT. Their breakdown strength under DC, AC, and 10 kHz voltage, tensile properties, dielectric response, surface potential decay, temperature-/electric field-dependent conductance, and field grading capability were investigated. Results show that loading BaTiO_3_ increases the dielectric constant and alters loss behavior due to enhanced interfacial polarization and modified charge transport. However, breakdown and tensile strengths decrease monotonically with filler content, which is attributed to interfacial heterogeneity and local field distortion. Shallow-trap density rises while trap energy level declines with higher BaTiO_3_ loading, promoting charge trapping–detrapping. Electrical conductivity of epoxy/BaTiO_3_ nanocomposites increases with both electric field and temperature, while simulation of electric field distribution in the triple point of IGBT encapsulation reveals that the increased permittivity and conductivity with BaTiO_3_ content can reduce the maximum local electric field by up to 6.7% and 13.7% for the two kinds of typical structure of triple points, respectively. Thus, nano-BaTiO_3_ effectively tailors dielectric response and charge transport but introduces interfacial complexity that degrades breakdown and mechanical performance. However, a trade-off between intrinsic insulation, tensile strength, and field grading capability can be obtained. This work offers experimental insights for designing epoxy-based encapsulation materials with tunable electrical properties for flexible DC systems.

## 1. Introduction

With the rapid development of flexible DC transmission technology and high-voltage, high-power power electronic equipment such as IGBTs, dry-type reactors, and wide-bandgap devices, encapsulation structures are subjected to higher voltage levels, higher power densities, and stronger electro-thermal coupled stresses [1,2,3]. Previous studies have shown that the reliability of wide-bandgap power electronic devices and their packaging structures is closely associated with thermal cycling, power cycling, packaging material failure, and interfacial degradation [1]. In high-voltage IGBT power modules, insulation failure modes such as partial discharge, electrical treeing, and water treeing can degrade encapsulation insulation systems, form conductive channels, and ultimately affect module reliability [2]. In addition, cast-resin dry-type epoxy insulation systems exhibit dielectric-response degradation, increased dielectric loss, and reduced breakdown performance after thermal or electro-thermal aging [3,4]. Conventional epoxy-based dry insulation also suffers from limited heat dissipation, which can intensify heat accumulation and insulation aging under high-power-density operation, thereby imposing stricter requirements on reliable encapsulation insulation materials [5].

Epoxy resin has long been one of the most widely used thermosetting matrices in electrical insulation and electronic packaging because of its excellent electrical insulation, high mechanical strength, good adhesion, and mature casting process [6]. However, neat epoxy still has inherent limitations, including low thermal conductivity, susceptibility to charge accumulation, and electrical conductivity and dielectric behavior that are sensitive to temperature and environmental conditions [7,8]. Under high electric fields and long-term operation, these factors may weaken insulation reliability. For encapsulation systems, material design should not focus only on high resistivity because local charge injection, transport, trapping, and dissipation also influence electric-field distribution and insulation lifetime [9]. Surface charge decay theory indicates that charge decay is closely related to both electrical conductivity and permittivity; therefore, designing insulating encapsulation materials with controllable conductivity has a clear physical basis [10]. The performance evolution of epoxy composite insulation under high-temperature and high-humidity conditions further highlights the importance of coordinated regulation of conductivity and dielectric parameters for high-voltage insulation applications [11].

In recent years, research on BaTiO_3_ has expanded beyond traditional high-permittivity fillers and ferroelectric ceramics toward emerging directions such as photocatalysis, electronic-structure modulation, heterostructure construction, and controlled synthesis. BaTiO_3_ has a typical perovskite structure, and its ferroelectric polarization, high dielectric constant, and tunable band structure provide important potential for polymer dielectric composites, energy storage, sensors, photocatalysis, and field-grading materials. Recent studies indicate that element fillering and composite heterostructures can further regulate the electronic structure, defect levels, and photogenerated carrier separation behavior of BaTiO_3_. For example, Kaptagay et al. investigated the effect of Rh fillering on the photocatalytic activity of BaTiO_3_ and showed that Rh fillering can introduce defect levels and regulate the oxygen evolution process, thereby improving photocatalytic activity [12]. Ivanov et al. prepared BaTiO_3_/CuO nanocomposites and studied their physicochemical properties and photocatalytic activity under visible and UV irradiation, showing that CuO incorporation can improve photogenerated charge separation and enhance photocatalytic efficiency under specific conditions [13]. Liu et al. tailored the aging time and calcination temperature in a sol–gel route to obtain highly tetragonal BaTiO_3_ nanoparticles, indicating that crystal phase, particle size, and processing route remain key factors governing the functional properties of BaTiO_3_ [14]. These studies show that the functionality of BaTiO_3_ can be finely designed through fillering, compositing, and structural regulation, providing an important material basis for conductivity regulation, interfacial polarization control, and local electric-field optimization in high-voltage insulation encapsulation materials.

It should be emphasized that research on epoxy/BaTiO_3_ composites for insulating encapsulation should not remain limited to enhancing the dielectric constant; greater attention must also be paid to their influence on charge transport and breakdown failure. Jia et al. investigated charge transport in epoxy/BaTiO_3_ composites at the interfacial scale and showed that local dynamic charge migration and trap characteristics at the filler/matrix interface significantly affect the overall conductivity and insulation performance of the material [15]. However, the introduction of BaTiO_3_ does not necessarily improve breakdown strength. Existing studies indicate that when the filler content increases, the interparticle distance decreases, or obvious agglomeration occurs, the composite becomes more prone to local electric-field enhancement, interfacial defects, and increased dielectric loss, thereby leading to a reduction in partial-discharge inception strength and breakdown strength [16,17]. Consistently, studies on breakdown enhancement in epoxy insulating composites have shown that the key to improving breakdown strength lies in the construction of controlled interfacial charge barriers and rational trap structures, rather than simply increasing filler loading [18].

Overall, existing studies on epoxy/BaTiO_3_ composites have focused predominantly on high dielectric response, energy-storage performance, and interfacial modification, whereas systematic studies on the intrinsic relationship among conductivity regulation, charge transport, and breakdown reliability under electrical encapsulation conditions remain insufficient. For core flexible DC components such as dry-type reactors and high-voltage, high-power semiconductor devices, the critical requirement for insulating encapsulation materials is not the extreme value of a single parameter, but a balanced combination of dielectric performance, conductive behavior, breakdown strength, thermal stability, and structural adaptability. Against this background, a systematic investigation of epoxy/BaTiO_3_ composites is of clear theoretical and engineering significance, particularly in clarifying how BaTiO_3_ content affects conductivity, breakdown strength, mechanical properties, dielectric spectra, and thermal behavior, and in elucidating the role of interfacial regulation in insulation reliability evolution.

## 2. Materials and Methods

### 2.1. Materials

The epoxy resin used in this study was E51 (LR grade, Shanghai Macklin Biochemical Co., Ltd., Shanghai, China). The curing agent was 4-methylhexahydrophthalic anhydride (MHHPA, 98%, LR grade, Shanghai Macklin Biochemical Co., Ltd., Shanghai, China). The accelerator was 2,4,6-tris(dimethylaminomethyl)phenol (DMP-30, 95%, Shanghai Meryer Chemical Technology Co., Ltd., Shanghai, China). The filler was nano barium titanate modified by KH560 coupling agent (BaTiO_3_, 99.9%, Beijing Deke Daojin Science And Technology Co., Ltd., Beijing, China).

### 2.2. Preparation of Epoxy/BaTiO_3_ Resin Nanocomposites

A predetermined amount of E51 epoxy resin was first added to a beaker. The curing agent MHHPA was then added according to the epoxy equivalent, and DMP-30 was introduced at 0.5 wt% of the total formulation. Surface-modified nano-BaTiO_3_ was added according to the designed composition to form the precursor mixture. The mixture was stirred using a high-speed mixer at 2000 r/min for 15 min, allowed to stand for 15 min for preliminary degassing, and then vacuum-degassed for 30 min to obtain a homogeneous casting slurry. The slurry was subsequently poured into pretreated molds and thermally cured in an oven. Based on DSC results, a stepwise curing schedule of 80 °C/2 h, 105 °C/2 h, and 120 °C/4 h was adopted. After curing, epoxy/BaTiO_3_ nanocomposite samples were obtained.

### 2.3. Experimental Methods

To systematically investigate the electrical, mechanical, dielectric, and thermal properties of the epoxy/BaTiO_3_ nanocomposites, breakdown strength, tensile performance, broadband dielectric spectroscopy, surface potential decay (SPD), thermal stability, and conduction behavior were characterized.

#### 2.3.1. Breakdown Strength Measurement

The breakdown strength of the composite samples was measured using AC and DC breakdown testing systems. Prior to testing, the samples were machined to the specified dimensions and their surfaces were cleaned to minimize the influence of surface defects and impurities. All breakdown samples used in this study were circular thin films with a thickness of 0.11 ± 0.02 mm. During testing, each sample was placed between ball electrodes and the breakdown voltage was recorded under continuous voltage ramping at a rate of 1 kV/s. During the test, each sample was placed between the upper and lower electrodes, and the breakdown voltage was recorded under continuous voltage ramping. AC and DC breakdown tests were conducted separately under their respective voltage waveforms to compare the insulation behavior of the composites under different electric-field conditions. Breakdown strength was calculated from the measured breakdown voltage and sample thickness. Multiple replicate tests were performed for each sample group to improve the reliability of the results.

#### 2.3.2. Tensile Testing

The mechanical properties of the samples were evaluated by tensile testing using a universal testing machine. Before testing, the samples were machined according to the standards of ISO 527 [19], with a narrow-section width of 10 mm, a preferred thickness of 4 mm, a gauge length of 75 mm, and a total length of 170 mm; smooth surfaces without obvious bubbles or cracks were ensured. During testing, the sample was clamped between the grips, and a uniaxial tensile load was applied at a loading rate of 20 mm/s. The load–displacement response was recorded to obtain tensile strength, elastic modulus, and elongation at break. Multiple tests were performed for each sample group, and the average values were used to evaluate the effect of nano-BaTiO_3_ incorporation on the mechanical performance of the epoxy matrix.

#### 2.3.3. Broadband Dielectric Spectroscopy

The dielectric properties of the samples were measured using a broadband dielectric spectroscopy system. Prior to testing, the samples were prepared as plate specimens suitable for parallel-plate electrode measurements, and good electrode contact was ensured on both sides. During testing, the complex dielectric response was measured over a prescribed frequency range to obtain the dielectric constant, dielectric loss, and AC conductivity at different frequencies. Analysis of these spectra was used to reveal the polarization behavior, interfacial relaxation characteristics, and frequency response of the nano-BaTiO3-filled epoxy composites.

#### 2.3.4. Surface Potential Decay (SPD) Measurement

To investigate surface charge accumulation and dissipation behavior, SPD measurements were carried out using a surface potential decay testing setup. During testing, the sample surface was first corona-charged under a specified voltage to generate an initial surface charge, and the surface potential was then continuously recorded as a function of time under zero applied electric field. By analyzing the decay curves and decay rates, the surface charge transport and dissipation capability of the composites could be evaluated, providing a basis for understanding conductivity regulation and interfacial trap characteristics.

#### 2.3.5. Conductivity Measurement

The conduction behavior of the samples was measured using a conductivity test system. Before testing, the samples were processed to the required dimensions; the samples used in this study were disks with a thickness of 0.11 mm and a diameter of 3 mm. Gold electrodes were deposited on the sample surfaces to ensure stable electrical contact. During testing, conduction current and resistance were measured under specified temperature and voltage conditions, and electrical conductivity was calculated. The conductivities of samples with different filler contents were compared to analyze the effect of nano-BaTiO_3_ on carrier transport and conduction behavior in the epoxy composites, providing experimental support for subsequent SPD and breakdown analyses.

#### 2.3.6. SEM and EDS Characterization

The fracture-surface morphology and filler dispersion state of neat epoxy and epoxy/BaTiO_3_ nanocomposites were characterized by scanning electron microscopy (SEM). Before observation, the cured samples were cryo-fractured in liquid nitrogen to obtain fresh fracture surfaces. The fracture surfaces were then sputter-coated with gold to improve surface conductivity and reduce charging during imaging. SEM images were used to observe fracture roughness, particle distribution, interfacial bonding, and local agglomeration in samples with different BaTiO_3_ contents.

To further analyze the elemental distribution of BaTiO_3_ in the epoxy matrix, energy-dispersive X-ray spectroscopy (EDS) mapping was performed on the fracture surfaces. Since Ba originates from the BaTiO_3_ filler, the Ba elemental distribution was selected as the main indicator for judging BaTiO_3_ dispersion. By comparing the uniformity, signal density, and local Ba-rich regions in the 10 wt%, 20 wt%, and 30 wt% BaTiO_3_/EP samples, the dispersion quality of BaTiO_3_ in the epoxy matrix was evaluated.

### 2.4. Simulation Methods

**Figure 1:**
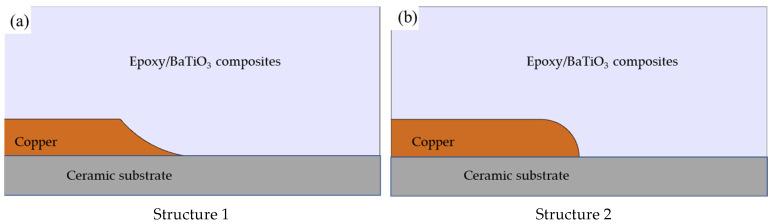
The model of typical triple points in encapsulation of IGBT for simulation of electric field distribution.

The electric field distribution characteristics at typical insulation weak points of high-voltage high-power IGBTs under different electrical conductivities and relative permittivities of epoxy/BaTiO_3_ nanocomposites were simulated and analyzed using the finite element method. The established finite element model in Comsol Multiphysics is shown in Figure 1, where two typical structures of triple points were considered. Both of the models consist of electrodes of copper electrode, encapsulation of epoxy/BaTiO_3_ nanocomposites, and ceramic substrate of alumina.

## 3. Results and Discussions

### 3.1. Breakdown Strength

**Figure 2:**
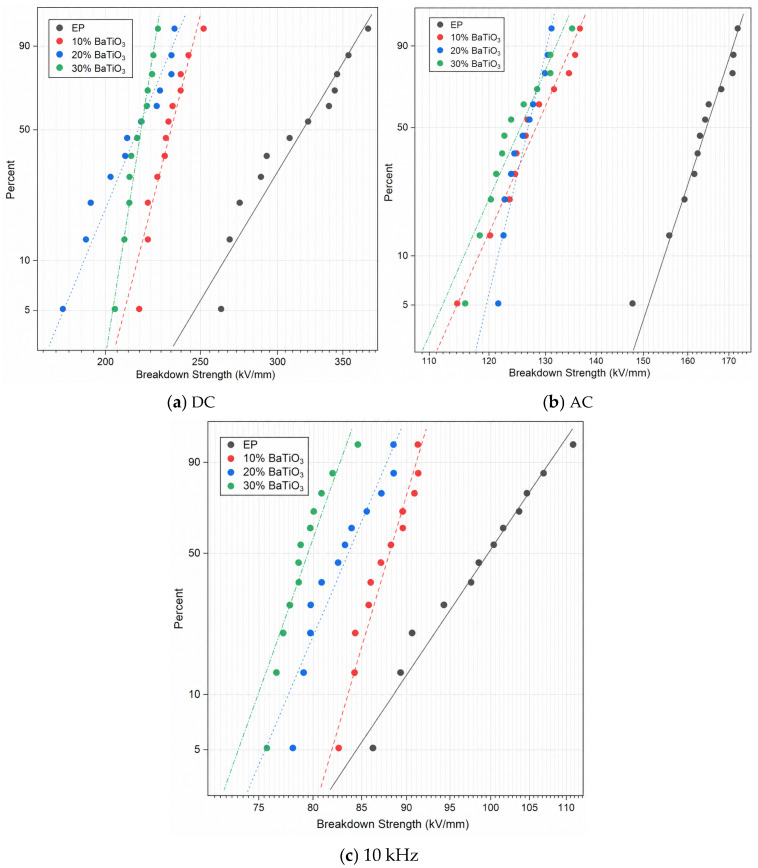
Weibull distributions of breakdown fields of epoxy/BaTiO_3_ nanocomposites at different voltage waveforms.

**Figure 3:**
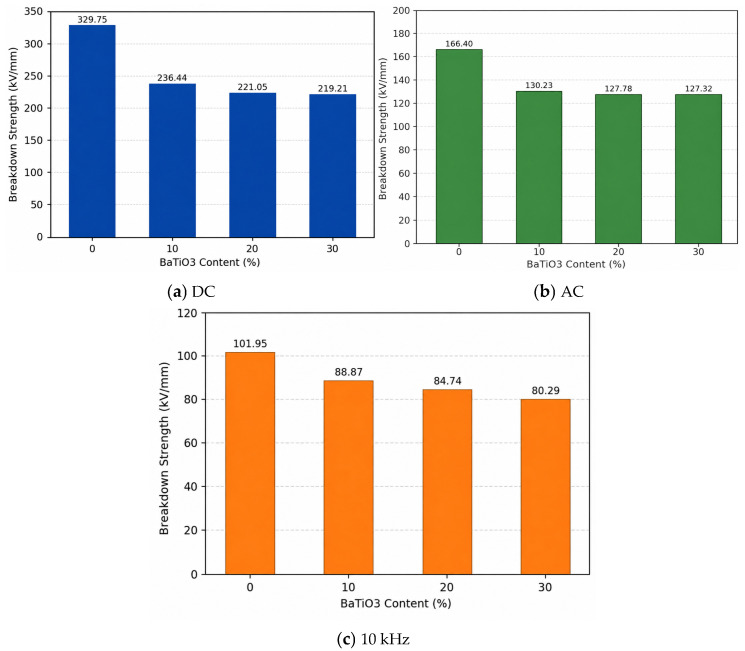
Breakdown strength of epoxy/BaTiO_3_ nanocomposites under different voltage waveforms.

The statistic results of the Weibull distribution are depicted in Figure 2, Table 1, and Figure 3, respectively. The results of breakdown strength under DC, AC, and 10 kHz conditions show that the breakdown strengths of BaTiO_3_-filled epoxy composites are lower than that of neat EP and generally decrease with increasing BaTiO_3_ content. Under DC conditions, the breakdown strength of neat EP is 329.75 kV/mm, whereas those of the 10, 20, and 30 wt% BaTiO_3_ samples decrease to 236.44, 221.05, and 219.21 kV/mm, respectively. Under AC conditions, the breakdown strength decreases from 166.4 kV/mm to 130.23, 127.78, and 127.32 kV/mm. Under 10 kHz conditions, it decreases from 101.95 kV/mm to 88.87, 84.74, and 80.29 kV/mm. These results indicate that, in this system, the introduction of BaTiO_3_ does not improve the breakdown performance of the epoxy matrix, but instead weakens breakdown strength to different degrees under all tested voltage forms.

This behavior is closely related to the pronounced interfacial effects in epoxy/BaTiO_3_ nanocomposites. Because of the large difference in dielectric constant between BaTiO_3_ and epoxy, introducing inorganic nanoparticles into an organic matrix forms many heterogeneous interfaces and changes the electric-field distribution around particles, leading to local field enhancement. According to the multi-core model and subsequent interface-oriented descriptions, bonded, bound, and loose layers exist around filler surfaces, and their electrical responses differ from both the ceramic core and the bulk matrix [20,21,22,23]. Charge injection, carrier trapping/detrapping, and local polarization therefore no longer occur uniformly in space, but become strongly interface-dominated. This effect is especially significant in epoxy/BaTiO_3_ systems because the high dielectric constant of BaTiO_3_ amplifies field nonuniformity at particle poles and narrow interparticle gaps, while the interphase can also become an important region for local charge accumulation and trap-assisted transport [21,23]. If the interphase does not form a sufficiently deep and spatially continuous charge-blocking barrier, accumulated charges may promote local electron multiplication, accelerate electrical tree initiation, and ultimately trigger premature breakdown.

Dispersion state is another key factor affecting breakdown. As BaTiO_3_ content increases, interparticle spacing decreases; if dispersion is insufficient, agglomerates and resin-occluded regions around them are more likely to form. Such heterogeneous microstructures are sensitive sites for increased local free volume, field crowding, and charge concentration, thereby promoting breakdown-channel propagation. Bell et al. demonstrated that filler dispersion and interfacial chemistry strongly influence dielectric breakdown in epoxy nanocomposites and emphasized that the beneficial role of nanofillers may be lost when designed interfaces are replaced by uncontrolled agglomeration [24]. Phan et al. further showed that epoxy matrix occlusions inside BaTiO_3_ aggregates alter local electrical responses, while Calabrese et al. reported that increased filler loading in polymer composites is often accompanied by increased dielectric loss and reduced dielectric strength [16,17]. Therefore, the continued decrease in breakdown strength for the 20 wt% and 30 wt% samples indicates that, in this material system, local defects and field concentration induced by dense interfacial regions and agglomeration outweigh the possible charge-barrier effect of the nanofillers.

### 3.2. Tensile Properties

**Figure 4:**
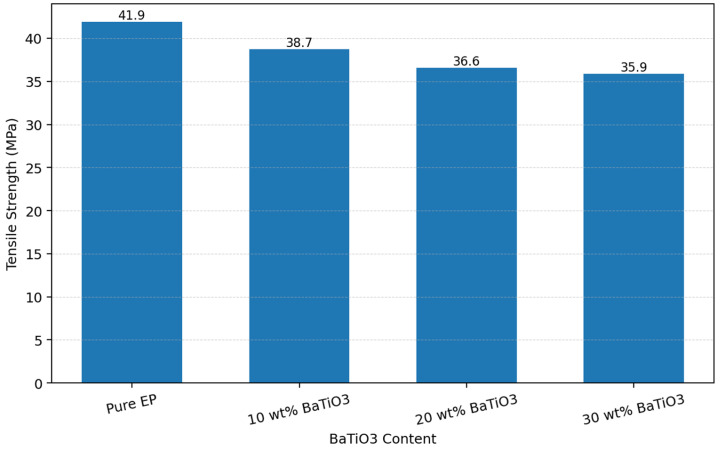
Tensile strength of samples with different BaTiO_3_ contents.

The tensile strength of epoxy/BaTiO_3_ nanocomposites with varied filler contents of BaTiO_3_ was depicted in Figure 4. As the BaTiO_3_ content increased from 0 wt% to 10, 20, and 30 wt%, the tensile strength of the composites decreased progressively from 41.9 MPa to 38.7, 36.6, and 35.9 MPa, respectively. This continuous decline indicates that the incorporation of BaTiO_3_ did not enhance the load-bearing capacity of the epoxy matrix, but instead weakened its tensile resistance. This behavior is mainly related to inadequate filler/matrix interfacial compatibility and the particle dispersion state. Because of the modulus mismatch between inorganic BaTiO_3_ particles and the organic epoxy matrix, the introduction of filler creates numerous heterogeneous interfaces, which can act as stress-concentration sites when interfacial bonding is insufficient. As the filler content increases, particle agglomeration and the associated microvoids or local defects become more likely, thereby impairing effective load transfer within the matrix and promoting crack initiation and propagation at the filler/matrix interface, which ultimately leads to a sustained decrease in tensile strength [25,26]. More generally, reviews on epoxy nanocomposites have emphasized that mechanical reinforcement depends not only on the intrinsic stiffness of the filler but also on whether the interphase can transfer stress effectively and suppress debonding under load [20,21]. In the present epoxy/BaTiO_3_ system, the observed monotonic reduction in tensile strength suggests that the interfacial region acts predominantly as a mechanically weak zone rather than a reinforcing bridge, and that the negative effects of agglomeration and stress concentration outweigh any stiffening contribution from the ceramic particles.

### 3.3. Broadband Dielectric Properties

**Figure 5:**
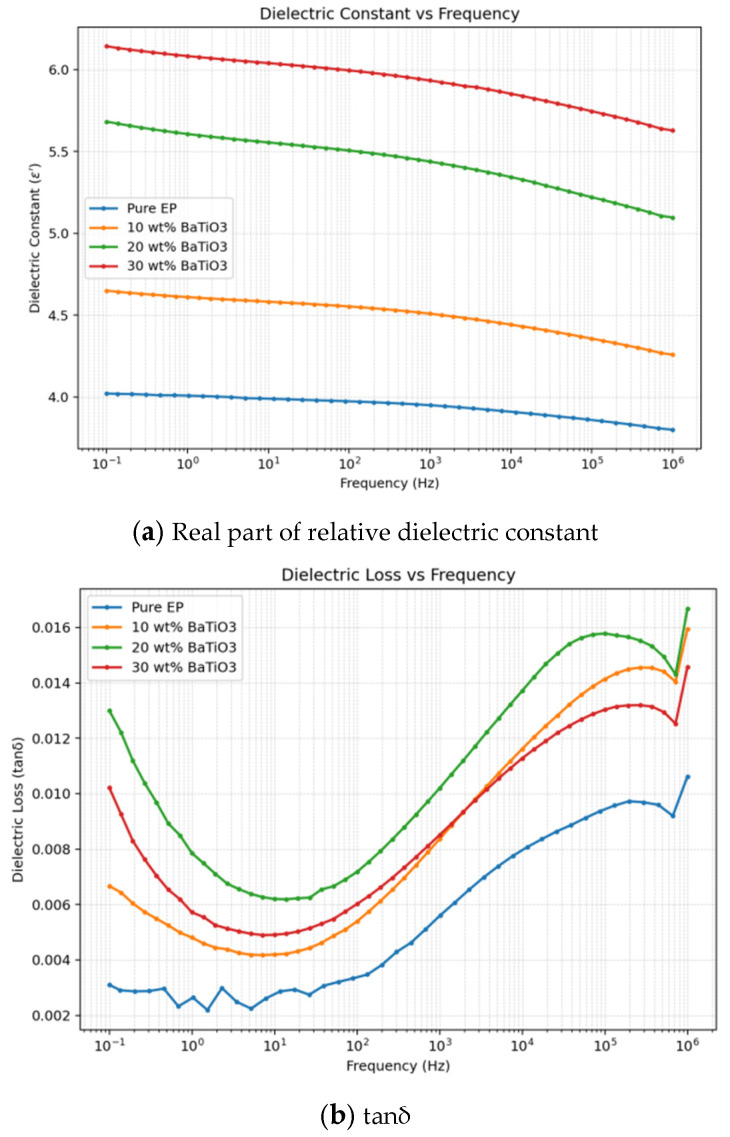
Frequency spectra of dielectric constant and dielectric loss for different samples.

Broadband dielectric spectroscopy in Figure 5 shows that neat EP exhibits relatively low dielectric constant and dielectric loss over the entire tested frequency range. After introducing BaTiO_3_, the dielectric constant of the composites increases significantly, and further increases as filler content increases from 10 wt% to 20 wt% and 30 wt%, indicating that the high dielectric response of BaTiO_3_ and interfacial polarization between BaTiO_3_ and the epoxy matrix jointly enhance the polarization capability of the system. Previous studies have shown that BaTiO_3_ content, dispersion state, and filler/matrix interfacial structure significantly affect the dielectric constant, dielectric loss, and relaxation behavior of epoxy/BaTiO_3_ composites [27,28,29]. Meanwhile, the dielectric constants of all samples gradually decrease with increasing frequency, indicating that dipolar orientation and interfacial charge accumulation have sufficient response time at low frequencies, whereas polarization units cannot follow the external field rapidly at high frequencies, leading to reduced effective polarization.

For dielectric loss, the 10 wt% and 20 wt% BaTiO_3_ samples show a frequency-dependent reversal trend. To avoid relying only on qualitative curve observation, representative tanδ values were extracted. In the low-frequency region, tanδ of the 20 wt% sample is higher than that of the 10 wt% sample; for example, at 0.1 Hz, 1 Hz, 10 Hz, and 100 Hz, tanδ values of the 10 wt% sample are 0.00667, 0.00481, 0.00419, and 0.00539, respectively, whereas those of the 20 wt% sample are 0.01022, 0.00572, 0.00491, and 0.00602. With increasing frequency, the difference gradually decreases, and a crossover tendency appears at approximately 2.14 kHz. In the high-frequency region, tanδ of the 10 wt% sample is slightly higher than that of the 20 wt% sample; for example, at 10 kHz, 100 kHz, and 1 MHz, tanδ values of the 10 wt% sample are 0.01162, 0.01414, and 0.01594, respectively, whereas those of the 20 wt% sample are 0.01127, 0.01303, and 0.01456. These results indicate that the dielectric-loss difference between the 10 wt% and 20 wt% samples is not a simple monotonic filler-loading effect, but has a clear frequency dependence.

This phenomenon can be understood as competition between low-frequency interfacial polarization/conduction loss and high-frequency localized relaxation loss. At low frequencies, the external field changes slowly, and carriers and dipoles have sufficient time to respond. Space charge can accumulate more readily at the BaTiO_3_/epoxy interfaces, giving rise to Maxwell–Wagner–Sillars (MWS) interfacial polarization and conductivity-related loss. MWS polarization generally originates from mismatch in permittivity and conductivity between different phases in a heterogeneous system and contributes significantly to dielectric constant and dielectric loss in the low-frequency region [30]. Because the 20 wt% sample contains more BaTiO_3_/epoxy interfaces and shorter interparticle spacing, interfacial charge accumulation and local conduction are stronger, so its low-frequency tanδ is higher than that of the 10 wt% sample. Ramajo et al. showed that introducing BaTiO_3_ particles changes the dielectric response and relaxation behavior of epoxy/BaTiO_3_ composites, and that these changes are closely related to filler content and interfacial polarization [27]. Drakopoulos et al. further indicated that dielectric response in epoxy/BaTiO_3_ nanodielectrics is jointly governed by matrix relaxation, interfacial polarization, and charge transport [21].

At high frequencies, the external field changes rapidly, so long-range space-charge migration and accumulation are suppressed. Dielectric loss is then more strongly controlled by localized dipolar relaxation, chain-segment motion near interfaces, and matrix polarization. Higher BaTiO_3_ content strengthens the restriction of inorganic particles on the motion of epoxy chain segments, making some dipoles and chain segments near interfaces unable to follow the high-frequency field, thereby reducing part of the high-frequency relaxation loss. Interface models of polymer nanodielectrics suggest that interfacial layers around nanofillers differ from the bulk matrix and can affect dipolar motion, trap distribution, carrier transport, and local conductivity [22,23]. Therefore, although the 20 wt% sample has more interfaces, chain-motion restriction and weakened localized relaxation reduce its high-frequency tanδ below that of the 10 wt% sample. The crossover tendency between the 10 wt% and 20 wt% samples reflects the competition between two mechanisms: at low frequencies, the 20 wt% sample is more strongly controlled by interfacial charge accumulation and dissipation, whereas at high frequencies the 10 wt% sample shows stronger localized relaxation loss.

It should further be noted that the 30 wt% sample does not show a second crossover similar to that between the 10 wt% and 20 wt% samples; instead, it maintains the highest dielectric loss over essentially the whole tested frequency range. For example, at 0.1 Hz, 10 Hz, 10 kHz, 100 kHz, and 1 MHz, tanδ values of the 30 wt% sample are 0.01300, 0.00620, 0.01371, 0.01578, and 0.01666, respectively, all higher than those of the 10 wt% and 20 wt% samples. This indicates that, when BaTiO_3_ content increases to 30 wt%, the number of interfaces, interparticle interactions, and local structural heterogeneity are significantly enhanced, while interfacial polarization, shallow-trap-assisted detrapping, local field distortion, and possible particle agglomeration jointly promote charge transport and energy dissipation. Jia et al. studied charge transport in BaTiO_3_/epoxy composites on an interfacial scale and showed that filler/matrix interfaces significantly affect local dynamic charge migration and macroscopic conduction behavior [15]. Although high filler loading in the 30 wt% sample still restricts epoxy chain motion, this effect is insufficient to offset the loss increase caused by abundant interfaces and enhanced charge transport. Therefore, the dielectric loss of the 30 wt% sample no longer exhibits a mechanism-competition-induced crossover as in the 10 wt% and 20 wt% samples, but is dominated by interfacial polarization and conduction-related loss across the full frequency range.

### 3.4. Surface Potential Decay and Trap Characteristics

To obtain the shallow-trap parameters of the samples, SPD curves were analyzed. During SPD testing, the samples were corona-charged and then the surface potential Vs(t) was recorded as a function of time t without external electric field. This decay process represents charge de-trapping, migration, and dissipation in the material, and therefore can be used to characterize trap features. In the data processing, the measured SPD curves were first fitted using a double-exponential function, as illustrated in Equation (1), to describe the charge dissipation processes at different time scales

(1) $$V_{s}(t)=a\cdot \exp(b\cdot t)+c\cdot \exp(d\cdot t).$$

According to the isothermal de-trapping model, the trap energy level *E*_T_ can be obtained from the decay time:

(2) $$E_{T}=k_{B}T\ln(v_{\mathrm{th}}t)$$

where *k*_B_ is the Boltzmann constant, *T* is testing temperature, and *v*_th_ is the effective de-trapping frequency.

The surface trapped charge density *Q*_s_ is calculated as:

(3) $$Q_{s}=-\frac{\varepsilon_{0}\varepsilon_{r}}{qL}t\frac{dV_{s}(t)}{dt}$$

where *ε*_0_ is vacuum permittivity, *ε_r_* is relative permittivity, *q* is element charge, and *L* is sample thickness. Furthermore, the trap density *N*_trap_ can be obtained as:

(4) $$N_{\mathrm{trap}}=-\frac{3Q_{s}}{\delta f}$$

where *δ* = 1.0 × 10^−6^ m and *f* = 0.5.

**Figure 6:**
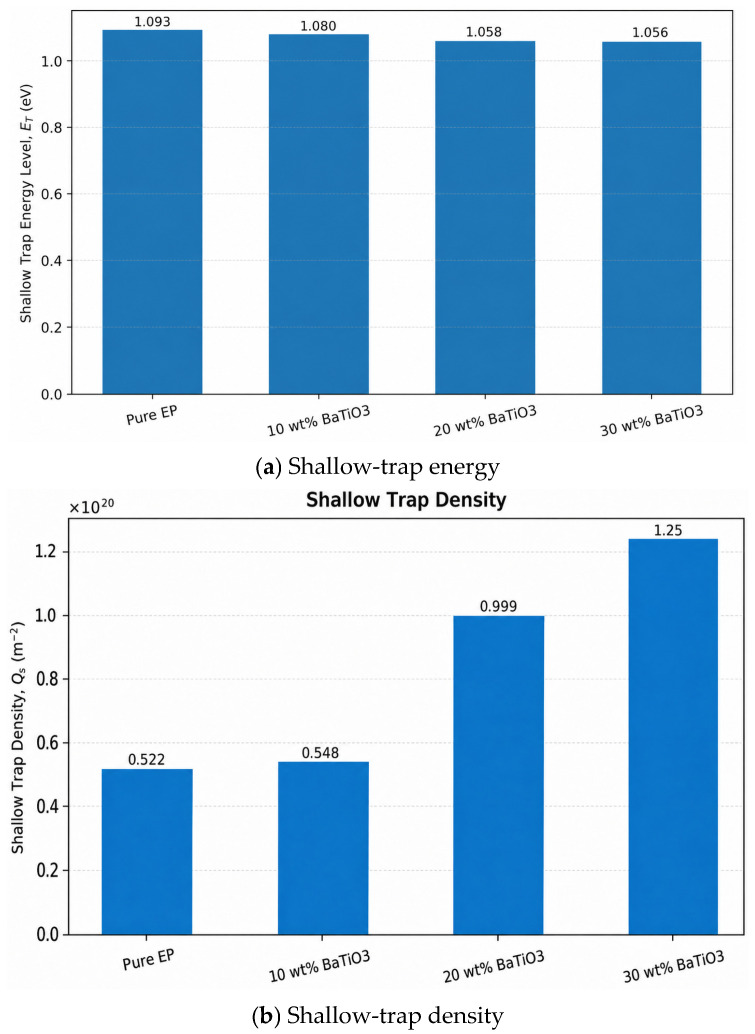
Shallow-trap energy level and shallow-trap density of different samples.

The shallow-trap results in Figure 6 show that, as BaTiO_3_ content increases from 0 wt% to 10, 20, and 30 wt%, shallow-trap density generally increases and reaches the maximum in the 30 wt% sample, while the shallow-trap energy level decreases from 1.093 eV to 1.056 eV. This indicates that BaTiO_3_ significantly changes the trap structure of epoxy. On the one hand, numerous heterogeneous filler/matrix interfaces increase the number of interfacial traps, thereby increasing shallow-trap density. On the other hand, as filler content increases, the extension of interfacial regions and enhanced interfacial interactions weaken the binding capability of some shallow traps, shifting the shallow-trap energy toward lower values. The simultaneous increase in shallow-trap density and decrease in trap energy imply that charges can undergo more frequent trapping–detrapping near interfaces, which favors interfacial polarization but is unfavorable for long-term suppression of charge migration [31,32]. From a broader interface-dominated nanodielectric model, the interphase may introduce a series of localized states rather than a single discrete trap level, and redistribution of shallow and deep traps can significantly affect dielectric response and electrical endurance [15]. Therefore, the observed trap evolution indicates that BaTiO_3_ gradually converts the epoxy matrix from a relatively uniform trapping environment into an interface-rich system, in which shallow and weakly bound localized states dominate short-range carrier motion.

### 3.5. Conductance Characteristics

**Figure 7:**
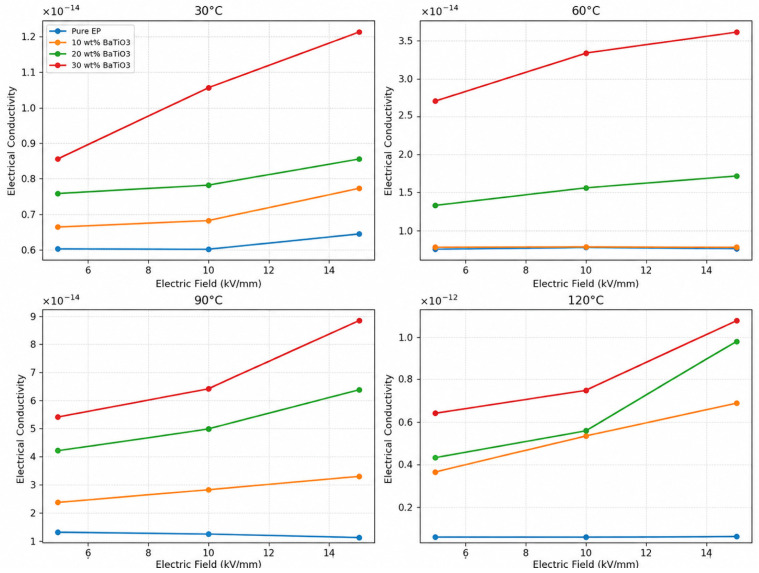
Variations in sample conductance with electric field at different temperatures.

At 30, 60, 90, and 120 °C, the conductivity of all samples continuously increases as the external electric field increases from 5 kV/mm to 15 kV/mm, indicating significant field-dependent conduction behavior, as shown in Figure 7. At the same electric field, conductivity also increases markedly with temperature, and the increase is more pronounced in the high-temperature region, indicating that temperature strongly promotes carrier migration and charge transport. In terms of filler content, increasing BaTiO_3_ content generally increases conductivity at all tested temperatures, and this difference becomes more obvious under high-temperature and high-field conditions. The 30 wt% sample usually exhibits the highest conductivity, while neat EP remains at a relatively low level. These trends indicate that the conduction process is jointly controlled by thermally activated carrier release, field-assisted transport, and interfacial regulation introduced by nanofillers [11].

This behavior arises from the coupling of thermally activated conduction, field-assisted detrapping, and interface-regulated conduction. With increasing temperature, polymer chain-segment mobility increases, allowing trapped carriers to acquire sufficient thermal energy for detrapping. Ion migration, hopping conduction, and thermally activated transport are therefore enhanced, resulting in increased conductivity. Previous studies have shown that the DC conductivity of epoxy resin and epoxy composite insulation exhibits clear temperature dependence and can be described by Arrhenius-type thermally activated behavior; at higher temperatures, ion migration and carrier release make important contributions to conductivity enhancement [33]. In epoxy encapsulation materials, conductivity extracted from dielectric spectra has also been reported to show hopping transport characteristics, with different activation energies in different temperature ranges, indicating that carrier hopping between localized states or across local barriers is an important conduction mode in epoxy systems [34].

Meanwhile, increasing the external electric field lowers the effective barrier for carrier escape from traps and promotes trap-assisted detrapping and migration. In the Poole–Frenkel emission mechanism, a strong electric field can lower the Coulombic trap barrier, making trapped carriers more easily thermally excited to conduction or transport states and leading to increased conductivity with increasing field [35]. Therefore, the increase in conductivity for all samples as the field increases from 5 kV/mm to 15 kV/mm is consistent with the basic features of field-assisted detrapping and Poole–Frenkel-type bulk conduction. Similarly, Akram et al. showed that the Poole–Frenkel effect has significant temperature dependence in polymer nanocomposite films, and increasing temperature increases the number of carriers thermally excited to trap-related transport states, thereby improving current conduction [36].

The introduction of BaTiO_3_ further enhances these processes. On the one hand, BaTiO_3_/epoxy interfaces introduce numerous localized states and interfacial traps, changing carrier trapping, detrapping, and migration paths. On the other hand, as filler content increases, the number of interfaces increases and interparticle spacing decreases, making charge accumulation and short-range hopping near interfaces more likely and thereby increasing the overall conductivity of the composites. Jia et al. showed that filler/matrix interfaces significantly affect local dynamic charge migration and macroscopic conductivity in BaTiO_3_/epoxy composites [15]. In addition, Alam et al. studied the conduction mechanisms of epoxy–BaTiO_3_ composite dielectrics and showed that leakage current behavior in polymer–BaTiO_3_ composite dielectrics is closely related to temperature, voltage, and aging conditions and should be analyzed using multiple mechanisms, including Schottky emission, Poole–Frenkel emission, and ion hopping [37]. The observed increase in conductivity with temperature, electric-field strength, and BaTiO_3_ content in this work can therefore be attributed to the combined effects of thermally activated carrier release, Poole–Frenkel-type field-assisted detrapping, hopping conduction, and BaTiO_3_/epoxy interface-mediated charge transport.

For the phenomenon that conductivity increases while breakdown strength decreases with increasing BaTiO_3_ content, the dominant mechanisms of macroscopic electric-field grading and high-field breakdown should be distinguished. Higher conductivity facilitates space-charge dissipation and, under long-term or quasi-steady operating conditions in power electronic encapsulation materials, can partly weaken field distortion caused by local charge accumulation, thus providing field-regulation capability. However, during breakdown testing, the material is under rapid voltage ramping and high electric-field stress. Breakdown is not determined only by whether charges can dissipate, but is also closely related to carrier injection, trap capture/detrapping, carrier mobility, and high-energy electron multiplication. According to electron avalanche and impact ionization breakdown theory, free electrons can gain sufficient energy in a strong electric field and generate secondary electrons through impact ionization, causing carrier multiplication. If this multiplication process continues in a local high-field region, a penetrating discharge channel can form and ultimately cause dielectric breakdown [27].

In the present system, as BaTiO_3_ content increases, the number of filler/matrix interfaces increases, shallow-trap density rises, and trap energy decreases, making carriers more likely to undergo trapping–detrapping cycles and interface-assisted migration. This improves apparent conductivity and surface charge decay capability, but also increases the number of electrons participating in conduction and impact ionization under high fields. When electrons acquire sufficient energy near locally enhanced fields at BaTiO_3_/epoxy interfaces, impact ionization and carrier multiplication become more likely. If this process develops along filler-agglomeration regions, interfacial defects, or local high-field regions, a penetrating discharge channel gradually forms, resulting in reduced breakdown strength. BaTiO_3_ therefore has a dual effect: at the macroscopic field-distribution level, increased dielectric constant and conductivity help reduce the maximum local electric field at the triple junction of IGBT encapsulation; at the intrinsic material-breakdown level, interfacial heterogeneity, shallow-trap-dominated carrier transport, and increased probability of impact ionization under high field promote discharge-channel formation, causing breakdown strength to decrease under DC, AC, and 10 kHz conditions.

### 3.6. The Simulation of Electric Field Distribution in IGBT Encapsulated by Epoxy/BaTiO_3_ Composites

**Figure 8:**
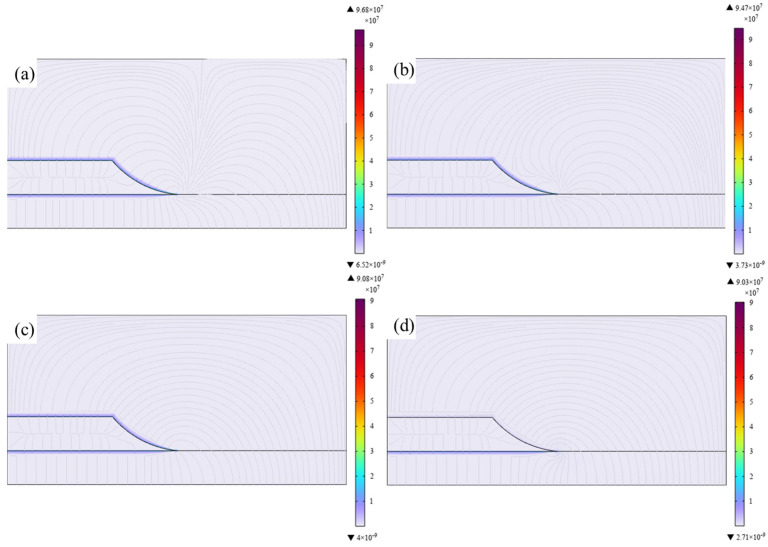
Electric field distribution in Structure 1 of triple points in encapsulation of IGBT when different epoxy/BaTiO_3_ nanocomposites with (**a**) 10^−15^ S/m, relative permittivity of 4, (**b**) 10^−14^ S/m, relative permittivity of 4.7, (**c**) 10^−13^ S/m, relative permittivity of 5.7, and (**d**) 10^−12^ S/m, relative permittivity of 6.2 were used.

**Figure 9:**
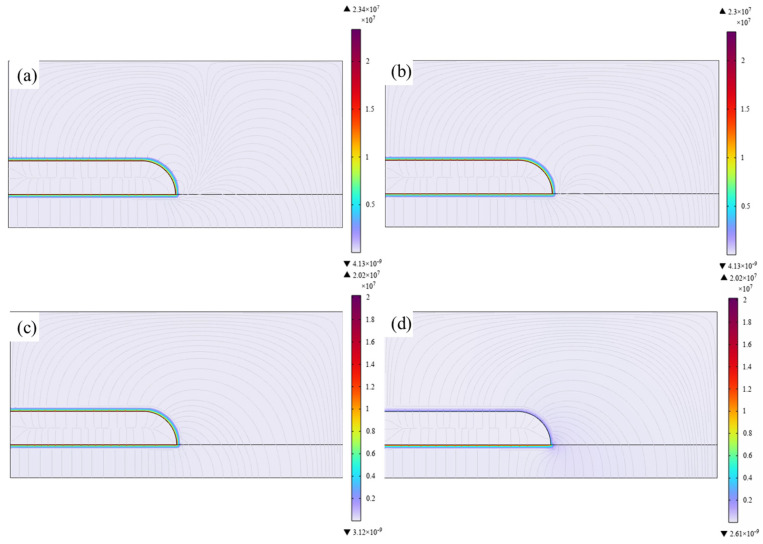
Electric field distribution in Structure 2 of triple points in encapsulation of IGBT when different epoxy/BaTiO_3_ nanocomposites with (**a**) 10^−15^ S/m, relative permittivity of 4, (**b**) 10^−14^ S/m, relative permittivity of 4.7, (**c**) 10^−13^ S/m, relative permittivity of 5.7, and (**d**) 10^−12^ S/m, relative permittivity of 6.2 were used.

The electric field distribution characteristics obtained by introducing the electrical conductivity and dielectric constant of different epoxy/BaTiO_3_ nanocomposites into the electric field simulation model for the triple junction weak point of the typical IGBT package structure are shown in Figure 8 and Figure 9, respectively. In this study, electrical conductivities of 1 × 10^−15^, 1 × 10^−14^, 1 × 10^−13^, and 1 × 10^−12^ S/m were adopted, while the corresponding relative permittivity were taken as 4, 4.7, 5.7, and 6.2, respectively, considering the range of electrical conductivity and relative permittivity tuned by the mass fraction of BaTiO_3_. It can be seen from Figure 8 that the maximum electric field intensities for Structure 1 are 96.8, 94.7, 90.8, and 90.2 kV/mm, respectively. Compared with the pure epoxy resin, the maximum electric field intensity decreases by 2.1%, 6.2%, and 6.7%, respectively. It is indicated by Figure 9 that the maximum electric field intensities for Structure 2 are 23.4, 23, 20.2, and 20.2 kV/mm, respectively. Compared with the pure epoxy resin, the maximum electric field intensity decreases by 1.7%, 13.7%, and 13.7%, respectively. Therefore, this study demonstrates that by doping with BaTiO_3_, the electrical conductivity and dielectric constant can be simultaneously tuned to reduce the local electric field distortion in power equipment, providing theoretical and experimental support for the development of encapsulation insulation materials for power equipment.

It should be noted that the simulation results in Figure 8 and Figure 9 mainly reflect the regulation of macroscopic electric-field distribution by different epoxy/BaTiO_3_ nanocomposites in the IGBT encapsulation geometry and cannot be directly equated with changes in intrinsic breakdown strength. In the simulation, maximum electric-field strength is jointly determined by relative permittivity, conductivity, and structural boundary conditions. With increasing BaTiO_3_ content, the permittivity and conductivity of the composites increase, partially alleviating field concentration near the triple junction. The degree of reduction in the maximum electric field strength varies with different electrode structures, which also indicates that the electric field distribution is greatly influenced by the structure, differing from the microscopic physical process of breakdown in epoxy/BaTiO_3_ composites [38]. This is because breakdown strength is not only determined by the macroscopic average field or the maximum field in insulation structure but is also sensitive to microscopic charge dynamics and defect structures inside the material. The DC breakdown model for polymer nanocomposites proposed by Li et al. indicates that DC breakdown involves electrode charge injection, carrier migration, deep-trap capture/de-trapping, charge recombination, and carrier energy acquisition in locally distorted fields [39]. Therefore, material breakdown is strongly local and dynamically evolving and cannot be explained solely by steady-state macroscopic field changes in finite-element simulations.

As a result, the simulation of electric field distribution and breakdown results of epoxy/BaTiO_3_ nanocomposites are not contradictory, but reflect the dual effect of BaTiO_3_ on encapsulation insulation materials. At the device scale, higher permittivity and conductivity help reduce macroscopic field concentration at the triple junction of IGBT encapsulation. At the material scale, interfacial heterogeneity, shallow-trap dominated carrier transport, local field distortion, and increased impact-ionization probability accelerate the microscopic formation of the breakdown channel, leading to a decrease in intrinsic breakdown strength.

### 3.7. SEM and EDS Distribution of Epoxy/BaTiO_3_ Composites

**Figure 10:**
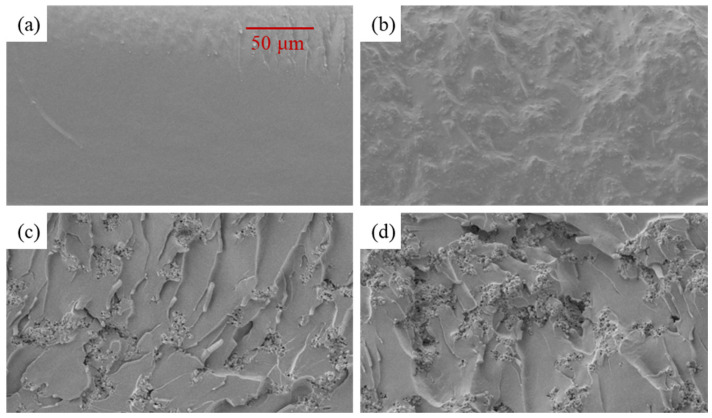
SEM images of different samples: (**a**) EP; (**b**) 10% BaTiO_3_; (**c**) 20% BaTiO_3_; (**d**) 30% BaTiO_3_.

As shown in Figure 10, SEM images show that the fracture surface of neat EP is generally smooth and compact, with only a few shallow scratches and slight local undulations, indicating that the epoxy matrix without BaTiO_3_ has a relatively uniform internal structure and fewer interfacial defects. In contrast, after introducing BaTiO_3_, the fracture surface becomes much rougher, with more tearing marks, lamellar fracture steps, and particle-like protrusions, indicating that inorganic fillers alter the fracture morphology and local stress distribution of the epoxy matrix.

For the 10 wt% BaTiO_3_/EP sample, relatively rough structures and local particle distributions begin to appear on the fracture surface, indicating that BaTiO_3_ forms filler/matrix interfaces in the epoxy matrix while overall agglomeration remains relatively low. When BaTiO_3_ content increases to 20 wt%, more particle-like regions and local agglomerates are observed, and obvious interfacial discontinuities and crack-deflection traces appear near the fillers. This indicates that increased filler content reduces interparticle spacing, increases the number of interfacial regions, and enhances microstructural heterogeneity. At 30 wt%, particle agglomeration and local pores/defects become more obvious, and filler-rich regions are more densely distributed, indicating that high BaTiO_3_ content more readily forms local agglomerates and introduces more interfacial defects and stress-concentration sites into the epoxy matrix.

**Figure 11:**
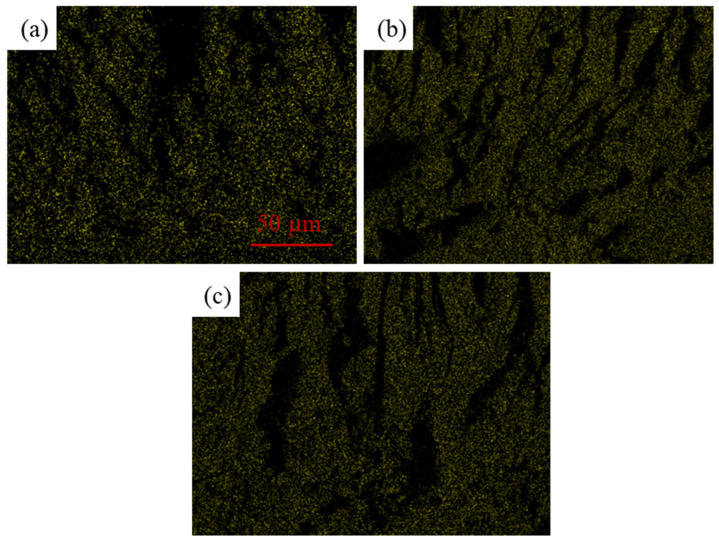
EDS images of different samples: (**a**) 10% BaTiO_3_; (**b**) 20% BaTiO_3_; (**c**) 30% BaTiO_3_.

EDS mapping of the Ba elemental further confirms the distribution of BaTiO_3_ in the epoxy matrix, which was illustrated in Figure 11. In the 10 wt% sample, the Ba signal is relatively dispersed, indicating that low-content BaTiO_3_ can be distributed relatively uniformly in the matrix. When the BaTiO_3_ content increases to 20 wt% and 30 wt%, the intensity and density of the Ba signal increase significantly, and local Ba-rich regions become more prominent, indicating a certain degree of local aggregation of BaTiO_3_ particles at high filler loading. The EDS results indicate that the dispersion quality of BaTiO_3_ in the cured composites gradually deteriorates with increasing filler content.

This microscopic dispersion state is consistent with the observed changes in material properties. On the one hand, introduction of BaTiO_3_ increases the filler/epoxy interfacial area, which helps enhance interfacial polarization and increase the dielectric constant. On the other hand, particle agglomeration, interfacial discontinuities, and local heterogeneity caused by high BaTiO_3_ content can induce local electric-field distortion and stress concentration, making breakdown channels and cracks more likely to propagate along interfaces or filler-rich regions. Therefore, SEM fracture morphology and Ba elemental EDS mapping provide microstructural evidence for explaining the decrease in breakdown strength and tensile strength at high BaTiO_3_ contents.

## 4. Conclusions

This study systematically investigates the effects of BaTiO_3_ nanoparticle doping on the electrical and mechanical properties of epoxy nanocomposites for high-voltage insulation applications. The main conclusions are as follows:

(1) The results show that BaTiO_3_ incorporation consistently degrades the breakdown strength of epoxy/BaTiO_3_ nanocomposites under DC, AC, and 10 kHz conditions, as well as the tensile strength, slightly. With 30 wt% BaTiO_3_, breakdown strength under DC, AC, and 10 kHz field drops from 329.75 to 219.21 kV/mm, from 166.4 to 127.32 kV/mm, and from 101.95 to 80.29 kV/mm, respectively. In addition, tensile strength falls from 41.9 to 35.9 MPa, indicating that filler-induced interfaces and agglomeration act as weak points rather than reinforcements, which can be explained by results of SEM and EDS.

(2) Loading BaTiO_3_ increases the dielectric constant (from ~4 to 6.2) and introduces a frequency-dependent loss crossover between 10 and 20 wt% samples. In addition, shallow-trap density rises while trap energy decreases (from 1.093 to 1.056 eV), favoring interfacial polarization but also facilitating charge migration. Electrical conductivity increases with temperature, electric field, and BaTiO_3_ content, where the sample epoxy/BaTiO_3_ with filler content of 30 wt% exhibits the highest conductance under all conditions, confirming thermally and field-assisted interfacial transport.

(3) Simulation of electric field distribution in triple point of IGBT encapsulation reveals that the increased permittivity and conductivity of epoxy/BaTiO_3_ nanocomposites with different BaTiO_3_ contents can reduce electric field distortion. In detail, the maximum local electric fields were reduced by up to 6.7% and 13.7% for two kinds of typical structure of triple-points, respectively, demonstrating a trade-off between intrinsic insulation, tensile strength, and field grading capability.

## Figures and Tables

**Table 1:** Parameters of Weibull distributions of breakdown field strength of epoxy/BaTiO_3_ nanocomposites under different voltage waveforms.

Testing Voltage Waveform	Sample	Shape	Scale	*N*	*p*
DC	EP	10.19	329.75	12	≥0.25
DC	10% BaTiO_3_	23.89	236.44	12	≥0.25
DC	20% BaTiO_3_	14.81	221.05	12	≥0.25
DC	30% BaTiO_3_	38.77	219.21	12	≥0.25
AC/50 Hz	EP	30.82	166.40	12	≥0.25
AC/50 Hz	10% BaTiO_3_	22.96	130.23	12	≥0.25
AC/50 Hz	20% BaTiO_3_	44.00	127.78	12	≥0.25
AC/50 Hz	30% BaTiO_3_	23.25	127.32	12	≥0.25
AC/10 kHz	EP	15.74	101.95	12	≥0.25
AC/10 kHz	10% BaTiO_3_	35.93	88.87	12	≥0.25
AC/10 kHz	20% BaTiO_3_	24.83	84.74	12	≥0.25
AC/10 kHz	30% BaTiO_3_	30.04	80.29	12	0.1813

## Data Availability

The original contributions presented in this study are included in the article. Further inquiries can be directed to the corresponding authors.

## References

[1] Wang Y., Ding Y., Yin Y. (2022). Reliability of Wide Band Gap Power Electronic Semiconductor and Packaging: A Review. Energies.

[2] Ciappa M. (2002). Selected failure mechanisms of modern power modules. Microelectron. Reliab..

[3] Dong Y., Wang Y., Li Z., Song B. (2026). Assessment of Insulation Aging Condition for Dry-Type Transformer Epoxy Resin Based on Dielectric Response and Activation Energy Analysis. Energies.

[4] Feng J.J., Zhang X., Wang W., Zhang W., Ren P., Peng P. Research on Thermal Aging Characteristics of Dry-type Transformer Epoxy Resin Based on Dielectric Response and Activation Energy. Proceedings of the 2019 IEEE Conference on Electrical Insulation and Dielectric Phenomena (CEIDP).

[5] Zhu Y.C., Qiu Z.B., Wu R.W. (2025). Thermal performance optimization of 10 kV dry-type transformer: A dual-strategy approach combining silicone rubber-enhanced insulation material and air gaps redesign. Case Stud. Therm. Eng..

[6] Dallaev R., Pisarenko T., Papež N., Sadovský P., Holcman V. (2023). A brief overview on epoxies in electronics: Properties, applications, and modifications. Polymers.

[7] He J., Wang H., Su Z., Guo Y., Tian X., Qu Q., Lin Y.L. (2019). Thermal conductivity and electrical insulation of epoxy composites with graphene-SiC nanowires and BaTiO_3_. Compos. Part A Appl. Sci. Manuf..

[8] Song Y.H., Yin L.J., Zhong S.L., Feng Q.K., Wang H.D., Zhang P.J., Xu H.P., Liang T., Dang Z.M. (2024). A processable high thermal conductivity epoxy composites with multi-scale particles for high-frequency electrical insulation. Adv. Compos. Hybrid Mater..

[9] Chen G., Xu Z., Zhang L. (2007). Measurement of the surface potential decay of corona-charged polymer films using the pulsed electroacoustic method. Meas. Sci. Technol..

[10] Navarro-Rodriguez M., Palacios-Lidon E., Somoza A.M. (2023). The surface charge decay: A theoretical and experimental analysis. Appl. Surf. Sci..

[11] Tian F.Q., Zhang L., Zhang J.L., Xiao P. (2017). Space charge and dielectric behavior of epoxy composite with SiO_2_-Al_2_O_3_ nano-micro fillers at varied temperatures. Compos. Part B Eng..

[12] Kaptagay G.A., Satanova B.M., Abuova A.U., Konuhova M., Zakiyeva Z.Y., Tolegen U.Z., Koilyk N.O., Abuova F.U. (2025). Effect of rhodium doping for photocatalytic activity of barium titanate. Opt. Mater. X.

[13] Ivanov K., Melnik E., Sirotkin N., Agafonov A. (2025). The physicochemical properties of BaTiO_3_/CuO nanocomposites and their photocatalytic activity under visible and UV light irradiation. Opt. Mater..

[14] Liu S., Sun Y.H., Zhang D., Yuan Y., Lu L., Jia X.P., Lin H.W., Zhang H.X. (2026). Tailoring the synthesis of highly tetragonal BaTiO_3_ nanoparticles by regulating aging time and calcination temperature using sol-gel route. Crystals.

[15] Jia B., Zhou J., Chen J., Zhang Z., Wang Y., Lv Z., Wu K. (2023). Interfacial Insight of Charge Transport in BaTiO_3_/Epoxy Composites. Nanomaterials.

[16] Phan T.T.M., Chu N.C., Luu V.B., Nguyen X.H., Martin I., Carriere P. (2016). The role of epoxy matrix occlusions within BaTiO_3_ nanoparticles on the dielectric properties of functionalized BaTiO_3_/epoxy nanocomposites. Compos. Part A Appl. Sci. Manuf..

[17] Calabrese R.E., Bury E., Haque F., Koh A., Park C. (2022). Effects of filler composition, loading, and geometry on the dielectric loss, partial discharge, and dielectric strength of polymer composites. Compos. Part B Eng..

[18] Yang K., Zhao Y., Liu X. (2024). Enhanced breakdown strength of epoxy composites by constructing dual-interface charge barriers at the micron filler/epoxy matrix interface. Compos. Part B Eng..

[19] (2025). Plastics—Determination of Tensile Properties—Part 2: Test Conditions for Moulding and Extrusion Plastics.

[20] Adnan M.M., Tveten E.G., Glaum J., Ese M.H.G., Hvidsten S., Glomm W., Einarsrud M.A. (2019). Epoxy-Based Nanocomposites for High-Voltage Insulation: A Review. Adv. Electron. Mater..

[21] Drakopoulos S.X., Wu J., Maguire S.M., Srinivasan S., Randazzo K., Davidson E.C., Priestley R.D. (2024). Polymer nanocomposites: Interfacial properties and capacitive energy storage. Prog. Polym. Sci..

[22] Tanaka T. (2005). Dielectric nanocomposites with insulating properties. IEEE Trans. Dielectr. Electr. Insul..

[23] Roy M., Nelson J.K., MacCrone R.K., Schadler L.S., Reed C.W., Keefe R., Zenger W. (2005). Polymer nanocomposite dielectrics—The role of the interface. IEEE Trans. Dielectr. Electr. Insul..

[24] Bell M., Krentz T., Nelson J.K., Schadler L., Wu K., Breneman C., Zhao S., Hillborg H., Benicewicz B. (2017). Investigation of dielectric breakdown in silica-epoxy nanocomposites using designed interfaces. J. Colloid Interface Sci..

[25] Atli A., Noyel J.P., Hajjar A., Antouly K., Lemaire E., Simon S. (2022). Exploring the mechanical performance of BaTiO3 filled HDPE nanocomposites: A comparative study of the experimental and numerical approaches. Polymer.

[26] Mishra R.K., Li D., Chianella I., Goel S., Lotfian S., Yazdani Nezhad H. (2023). Low electric field induction in BaTiO_3_-epoxy nanocomposites. Funct. Compos. Mater..

[27] Ramajo L., Reboredo M.M., Castro M.S. (2005). Dielectric response and relaxation phenomena in composites of epoxy resin with BaTiO_3_ particles. Compos. Part A Appl. Sci. Manuf..

[28] Ramajo L., Castro M.S., Reboredo M.M. (2007). Effect of silane as coupling agent on the dielectric properties of BaTiO_3_–epoxy composites. Compos. Part A Appl. Sci. Manuf..

[29] Drakopoulos S.X., Psarras G.C. (2022). Epoxy-based/BaTiO_3_ nanodielectrics: Relaxation dynamics, charge transport and energy storage. Mater. Res. Bull..

[30] Sun H., Zhang H., Liu S., Ning N., Zhang L., Tian M. (2018). Interfacial polarization and dielectric properties of aligned carbon nanotubes/polymer composites: The role of Maxwell-Wagner-Sillars effect. Compos. Sci. Technol..

[31] Aslam F., Qu G., Feng Y., Li S. (2023). Electrical breakdown strength and interfacial trap characteristics of Epoxy/POSS nanocomposites. Org. Electron..

[32] Aslam F., Qu G., Feng Y., Li S. (2021). Improvement of DC Breakdown Strength of the Epoxy/POSS Nanocomposite by Tailoring Interfacial Electron Trap Characteristics. Materials.

[33] Tian F., Lei Q., Wang X., Wang Y. (2022). Effect of temperature on the charge transport behavior of epoxy resin and epoxy/nano-SiO_2_/micro-BN composite. Nanomaterials.

[34] Cornigli D., Raso G., Schijve W., Montanari G.C., Cavallini A. (2018). Characterization of dielectric properties and conductivity in encapsulation materials with high insulating filler contents. IEEE Trans. Dielectr. Electr. Insul..

[35] O’Dwyer J.J. (1973). The Theory of Electrical Conduction and Breakdown in Solid Dielectrics.

[36] Akram S., Khan M.T.A., Ashraf A., Khan A., Ziad H.M., Basyooni M.A., ElKabbash M., Ahmad I. (2023). Examining the mechanism of current conduction at varying temperatures in polyimide nanocomposite films. Energies.

[37] Alam M.A., Azarian M.H., Osterman M., Pecht M.G. (2011). Temperature and voltage aging effects on electrical conduction mechanism in polymer-BaTiO_3_ composite dielectric used in embedded capacitors. Microelectron. Reliab..

[38] Li S., Min D., Wang W., Chen G. (2016). Linking traps to dielectric breakdown through charge dynamics for polymer nanocomposites. IEEE Trans. Dielectr. Electr. Insul..

[39] Huan T.D., Boggs S., Teyssedre G., Laurent C., Cakmak M., Kumar S., Ramprasad R. (2016). Advanced polymeric dielectrics for high energy density applications. Prog. Mater. Sci..

